# Supplementary material for: QUIRMIA—A Phenotype-Based Algorithm for the Inference of Quinolone Resistance Mechanisms in Escherichia coli
Source: Antibiotics (Basel). 2023 Jun 28;12(7):1119. doi: 10.3390/antibiotics12071119 (PMC10376670; doi:10.3390/antibiotics12071119)
Supplement: Supplementary file 1 [file antibiotics-12-01119-s001.zip › antibiotics-2422110-supplementary.pdf]

Supplementary Materials

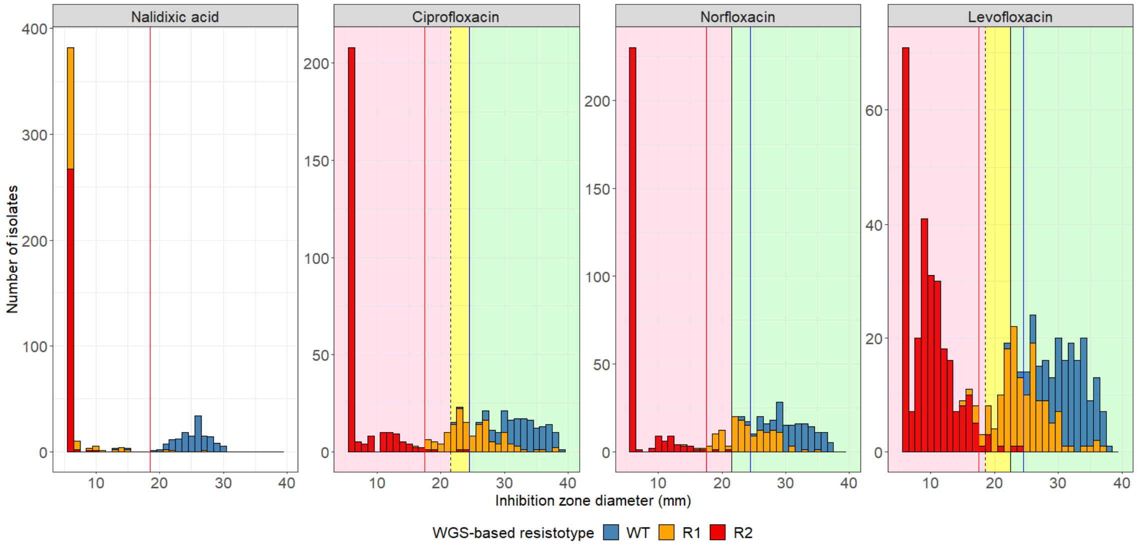

Figure S1. Distribution of the inhibition diameters with WGS-based clinical categorization. EUCAST lower and upper clinical breakpoints are indicated by the dashed and continous black lines, respectively. EUCAST ECOFFs and IMM working cut-offseparators are indicated by the blue and red continous lines, respectively. The coloured areas reflect the EUCAST clinical categories of susceptible (green), suscpetible by increased exposure (yellow) and resistant (red). WT, wild-type; R1, Low-level resistance; R2, High-level-resistance.

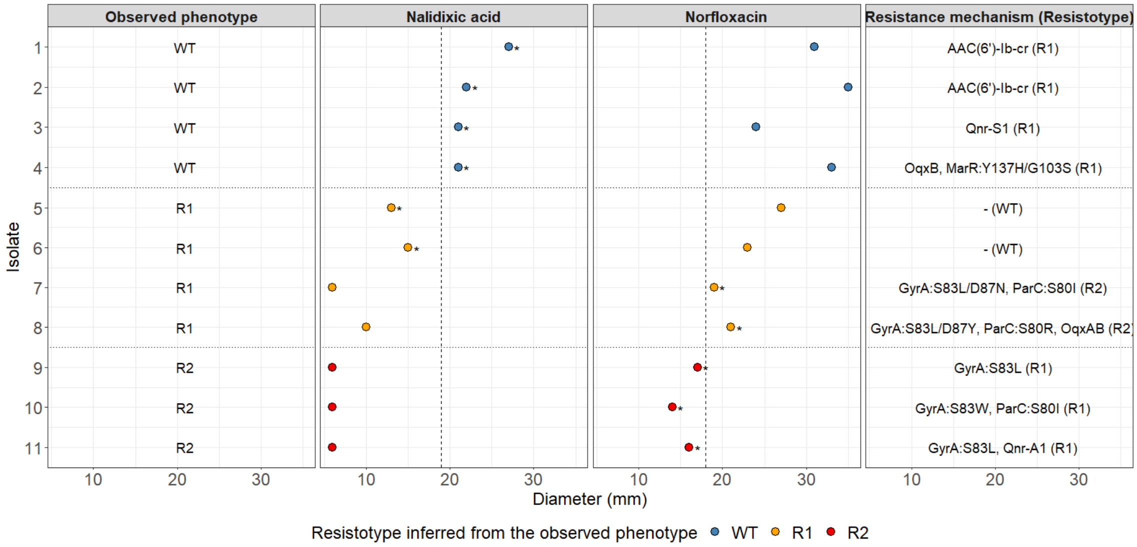

Figure S2. Inhibition zone diameters of isolates with discordant phenotype/genotype. Dots indicate the inhibition zone diameters of isolates with discrepant QUIRMIA-derived resistance mechanism(s) and WGS-based genotype(s). Asterisks indicate the inhibition diameter responsible for the discrepancy, respectively. Vertical dashed lines indicate IMM working cut-off separators. WT, wildtype; R1, low-level resistance, R2, high-level resistance.

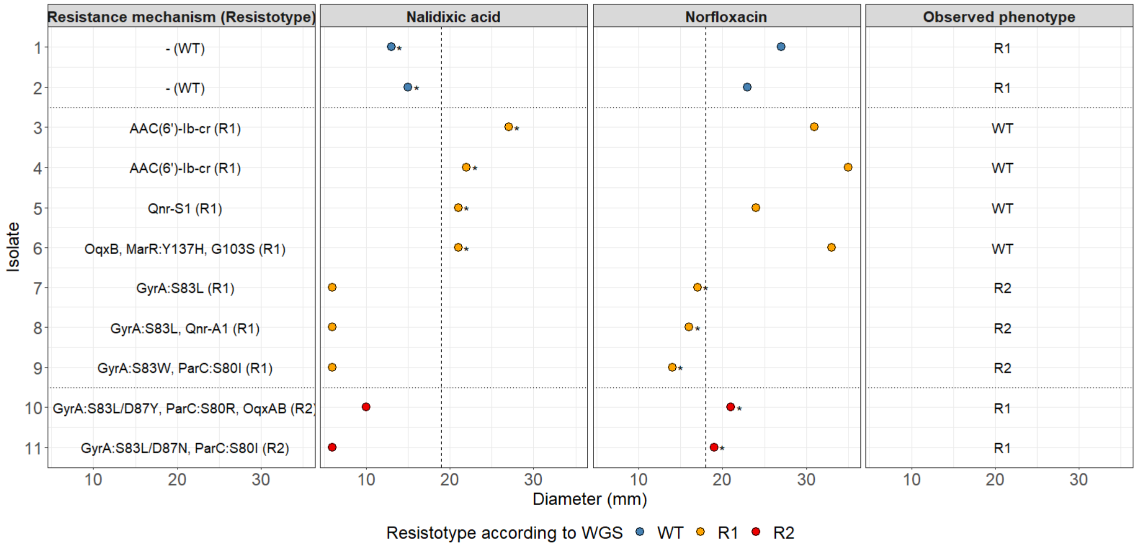

Figure S3. Inhibition zone diameters of isolates with discordant genotype/phenotype. Dots indicate the inhibition diameters of isolates with discrepancies regarding WGS-based predicted vs. observed phenotypes. Asterisks indicate the inhibition diameters responsible for the discrepancy, respectively. Vertical dashed lines indicate the IMM working cut-off separators. WT, wildtype; R1, low-level resistance, R2, high-level resistance.

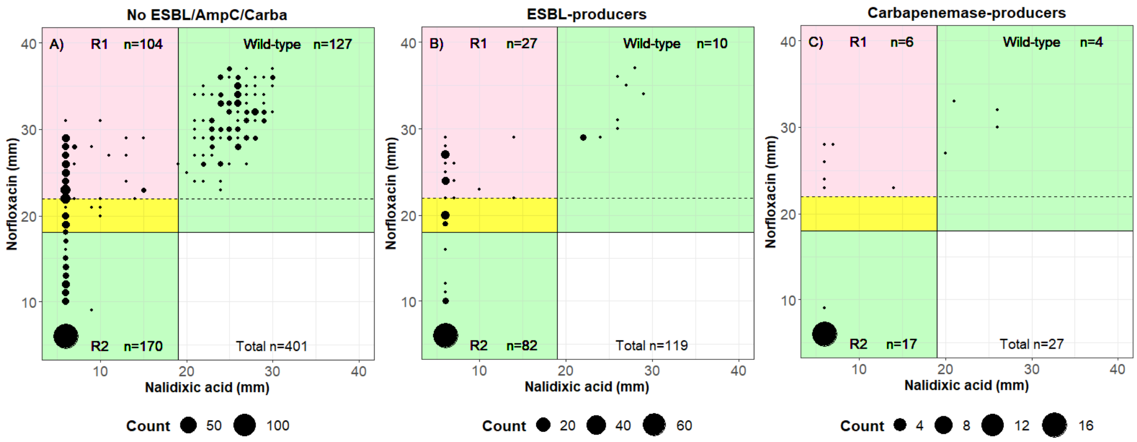

Figure S4. Inhibition zone diameter distributions of isolates grouped by β-lactamase production. Dots correspond to inhibition zone diameters for nalidixic acid and norfloxacin of A) isolates producing no ESBL or carbapenemases, B) ESBL-producers and C) carbapenemase-producers. Vertical and horizontal continous lines indicate the IMM working cut-off separators for norfloxacin and nalidixic acid, respectively. Horizontal dashed lines indicate the EUCAST ECOFFs for norfloxacin. Green backgrounds reflect concordance between EUCAST- and WGS-based classification. Red and yellow backgrounds reflect isolates classified as low-level resistant based on WGS data and as susceptibile or resistant according to EUCAST guidelines, respectively. R1, low-level resistance, R2, high-level resistance.

Table S1. List of fluoroquinolone resistance mechanisms in *Escherichia coli*.

**Resistance mechanism(s)**	**Mechanism of action**	**Location of the gene(s)**	**Resistance phenotype**
qnr [1-3]	Target protection	Plasmidic	Low-level resistance
AAC(6')-Ib-cr [4]	Drug inactivation	Plasmidic	Low-level resistance
marR mutations [5,6]	Drug efflux/descreased uptake	Chromosomal	Low-level resistance
acrR mutation [5,7]	Drug efflux	Chromosomal	Low-level resistance
soxS mutation [8]	Drug efflux	Chromosomal	Low-level resistance
oqxAB [9]	Drug efflux	Plasmidic	Low-level resistance
qep [10]	Drug efflux	Plasmidic	Low-level resistance
1 *gyrA* mutation [11]	Target modification	Chromosomal	Low-level resistance
1 *gyrA* mutation + 1 *parC* mutation [11]	Target modification	Chromosomal	Low-level resistance
2 *gyrA* mutations [11]	Target modification	Chromosomal	Low-level resistance
1 *gyrB* mutation [12]	Target modification	Chromosomal	Low-level resistance
2 *gyrA* mutations + 1 *parC* mutation [11]	Target modification	Chromosomal	High-level resistance

References

1. Briales A, Rodriguez-Martinez JM, Velasco C et al. In vitro effect of *qnrA1, qnrB1*, and *qnrS1* genes on fluoroquinolone activity against isogenic *Escherichia coli* isolates with mutations in *gyrA* and *parC*. *Antimicrob Agents Chemother* 2011; **55**: 1266-9.
2. Garoff L, Yadav K, Hughes D. Increased expression of Qnr is sufficient to confer clinical resistance to ciprofloxacin in *Escherichia coli*. *J Antimicrob Chemother* 2018; **73**: 348-52.
3. Jiang X, Li J, Zhang Y et al. Detection of plasmid-mediated quinolone resistance determinants and *qnrS* expression in *Enterobacteriaceae* clinical isolates. *J Infect Dev Ctries* 2014; **8**: 1625-9.
4. Robicsek A, Strahilevitz J, Jacoby GA et al. Fluoroquinolone-modifying enzyme: a new adaptation of a common aminoglycoside acetyltransferase. *Nat Med* 2006; **12**: 83-8.
5. Huseby DL, Pietsch F, Brandis G et al. Mutation Supply and Relative Fitness Shape the Genotypes of Ciprofloxacin-Resistant *Escherichia coli*. *Mol Biol Evol* 2017; **34**: 1029-39.
6. Praski Alzrigat L, Huseby DL, Brandis G et al. Fitness cost constrains the spectrum of marR mutations in ciprofloxacin-resistant *Escherichia coli*. *The Journal of antimicrobial chemotherapy* 2017; **72**: 3016-24.
7. Wang H, Dzink-Fox JL, Chen M et al. Genetic characterization of highly fluoroquinolone-resistant clinical *Escherichia coli* strains from China: role of *acrR* mutations. *Antimicrob Agents Chemother* 2001; **45**: 1515-21.
8. Aly SA, Boothe DM, Suh SJ. A novel alanine to serine substitution mutation in SoxS induces overexpression of efflux pumps and contributes to multidrug resistance in clinical *Escherichia coli* isolates. *J Antimicrob Chemother* 2015; **70**: 2228-33.
9. Hansen LH, Jensen LB, Sorensen HI et al. Substrate specificity of the OqxAB multidrug resistance pump in *Escherichia coli* and selected enteric bacteria. *J Antimicrob Chemother* 2007; **60**: 145-7.
10. Yamane K, Wachino J, Suzuki S et al. New plasmid-mediated fluoroquinolone efflux pump, QepA, found in an Escherichia coli clinical isolate. *Antimicrob Agents Chemother* 2007; **51**: 3354-60.
11. van der Putten BCL, Remondini D, Pasquini G et al. Quantifying the contribution of four resistance mechanisms to ciprofloxacin MIC in *Escherichia coli*: a systematic review. *The Journal of antimicrobial chemotherapy* 2019; **74**: 298-310.
12. Herrera G, Aleixandra V, Urios A et al. Quinolone action in *Escherichia coli* cells carrying *gyrA* and *gyrB* mutations. *FEMS microbiology letters* 1993; **106**: 187-91.

Table S2. QUIRMIA-based resistance phenotypes vs. genotypes

Phenotypic-based resistotype	Observed Phenotype	N. of isolates	Phenotype/ genotype concordance (n, %)	Phenotype/ genotype discordance (n, %)	Resistance mechanism(s) (N. of isolates) (in bold the discrepants)	Discrepant number	Predicted Phenotype	Clinical impact
**Wild-type (WT)**	NAL ≥ 19 mm	142	138 (97.2)	4 (2.8)	No resistance mechanism (138)		NOR^S	
	NOR ≥ 18 mm				**AAC(6')-Ib-*cr* (2)**	**1,2**	CIP^S	**mE**
					**Qnr-S1 (1)**	**3**	LEV^S	**mE**
					**OqxB + 2 *marR* mutations (1)**	**4**		**mE**
**Low-Level Resistance (R1)**	NAL < 19 mm	139	135 (97.1)	4 (2.9)	1 *gyrA* mutation (100)		NOR^I	
	NOR ≥ 18 mm				1 *gyrA* mutation + 1 *parC* mutation (8)		CIP^I	
					1 *gyrA* mutation + Qnr-S1 (5)		LEV^I	
					1 *gyrA* mutation + Qnr-B4 (3)			
					1 *gyrA* mutation + AAC(6')-Ib-*cr* (1)			
					1 *gyrA* mutation + 2 *marR* mutations (5)			
					Qnr-S1 (5)			
					Qnr-S1+ 2 *marR* mutations (1)			
					Qnr-S2 (2)			
					Qnr-B19 (1)			
					2 *gyrA* mutations (4)			
					**No resistance mechanism (2)**	5,6		-
					**2 *gyrA* mutations + 1 *parC* mutation (1)**	**7**		**mE**
					**2 *gyrA* mutations + 1 *parC* mutation + OqxAB (1)**	**8**		**mE**
**High-level resistance (R2)**	NAL < 19 mm	272	269 (98.9)	3 (1.2)	2 *gyrA* mutations + 1 *parC* mutation (108)		NOR^R	
	NOR < 18 mm						CIP^R	

	2 *gyrA* mutations + 1 *parC* mutation + 1 *gyrB* mutation + 2 *marR* mutations (1)		LEV^R	
	2 *gyrA* mutations + 1 *parC* mutation + AAC(6’)-Ib-*cr* (21)			
	2 *gyrA* mutations + 1 *parC* mutation + AAC(6’)-Ib-*cr* + 1 *marR* mutation (1)			
	2 *gyrA* mutations + 1 *parC* mutation + OqxAB (1)			
	2 *gyrA* mutations + 1 *parC* mutation + OqxAB + 2 *marR* mutations (1)			
	2 *gyrA* mutations + 1 *parC* mutation + Qep-A2 (1)			
	2 *gyrA* mutations + 1 *parC* mutation + Qep-A4 (1)			
	2 *gyrA* mutations + 1 *parC* mutation + Qnr-B4 (2)			
	2 *gyrA* mutations + 1 *parC* mutation + Qnr-S1 (4)			
	2 *gyrA* mutations + 1 *parC* mutation + Qnr-S1 + 2 *marR* mutations (1)			
	2 *gyrA* mutations + 1 *parC* mutation + 1 *marR* mutations (2)			
	2 *gyrA* mutations + 1 *parC* mutation + 2 *marR* mutations (10)			
	2 *gyrA* mutations + 1 *parC* mutation + 1 *soxR* mutation (1)			
	2 *gyrA* mutations + 1 *parC* mutation + Qnr-S2 (1)			
	2 *gyrA* mutations + 2 *parC* mutations (69)			
	2 *gyrA* mutations + 2 *parC* mutations + 1 *marR* mutation (2)			
	2 *gyrA* mutations + 2 *parC* mutations + 2 *marR* mutations (2)			
	2 *gyrA* mutations + 2 *parC* mutations + 1 *acrR* mutation (3)			
	2 *gyrA* mutations + 2 *parC* mutations + AAC(6’)-Ib (1)			
	2 *gyrA* mutations + 2 *parC* mutations + AAC(6’)-Ib-*cr* (34)			
	2 *gyrA* mutations + 2 *parC* mutations + AAC(6’)-Ib + OqxAB (1)	**9**		-
	2 *gyrA* mutations + 2 *parC* mutations + Qnr-D2 + 2 *marR* mutations (1)	**10**		-
	**1 *gyrA* mutation (1)**	**11**		-
	**1 *gyrA* mutation + 1 *parC* mutation (1)**			

				**1 *gyrA* mutation + QnrA1 (1)**		
**Total**	**553**	**542 (98)**	**11 (2)**		**11**	**6 mE**

N, number; GT, Genotype; PT, Phenotype

Table S3. Discrepancy table: QUIRMIA-based classification

Discrepant number	ST	NAL (mm)	NOR (mm)	CIP (mm)	LEV (mm)	Resistance mechanism(s)	QUIRMIA-based resistotype	WGS-based resistotype
1	141	27	31	**33**	**34**	*aac*(6')-Ib-cr	Wild-type	Low-level resistance
2	ND	22	35	**38**	**36**	*aac*(6')-Ib-cr	Wild-type	Low-level resistance
3	1421	21	24	**24**	**25**	*qnr*S1	Wild-type	Low-level resistance
4	ND	13	27	**23**	**22**	-	Low-level resistance	Wild-type
5	336	15	23	**22**	**25**	-	Low-level resistance	Wild-type
6	ND	6	22	**23**	**23**	-	Low-level resistance	Wild-type
7	162	6	19	**18**	**19**	*gyrA* S83L/D87N, *parC* S80I	Low-level resistance	High-level resistance
8	ND	10	21	**19**	**17**	*gyrA* S83L/D87Y, *parC* S80R, *oqxAB*	Low-level resistance	High-level resistance
9	131	6	17	**20**	**21**	*gyrA* S83L	High-level resistance	Low-level resistance
10	73	6	14	**18**	**20**	*gyrA* S83L, *parC* S80I	High-level resistance	Low-level resistance
11	ND	6	16	**18**	**16**	*gyrA* S83L, *qnrA1*	High-level resistance	Low-level resistance

ST, Sequence type; ND, not determined; NAL, Nalidixic acid (30 µg); NOR, Norfloxacin (10 µg); CIP, Ciprofloxacin (5 µg); LEV, Levofloxacin (5 µg); mE, minor Error; vME, very major error.

The phenotypes responsible for the discrepancies are highlighted in red.

Inhibition zone diameters highlighted in black bold were included for completion but were not used for the WGS classification.

Table S4. EUCAST-based classification vs. genotypes

Observed Phenotype	EUCAST-based catego-rization	N. of iso-lates	Resistance mechanism(s) (N. of isolates)	WGS-based classification
**CIP ≥ 25 mm**	Susceptible	207	No resistance mechanism (138)	Wild-type (138)
			1 *gyrA* mutation (52)	Low-level resistance (69)
			2 *gyrA* mutations (1)	
			1 *gyrA* mutation + 1 *parC* mutation (2)	
			1 *gyrA* mutation + Qnr-S1 (1)	
			1 *gyrA* mutation + Qnr-B4 (2)	
			1 *gyrA* mutation + 2 *marR* mutations (5)	
			AAC(6′)-Ib-*cr* (2)	
			Qnr-B19 (1)	
			Qnr-S1 (1)	
			Qnr-S1+ 2 *marR* mutations (1)	
			OqxB + 2 *marR* mutations (1)	
**CIP < 25 mm**	Resistant	346	No resistance mechanism (2)	Wild-type (2)
			1 *gyrA* mutation (49)	Low-level resistance (73)
			2 *gyrA* mutations (3)	
			1 *gyrA* mutation + 1 *parC* mutation (7)	
			1 *gyrA* mutation + Qnr-S1 (4)	
			AAC(6′)-Ib-*cr* (1)	

	Qnr-A1 (1)	
	Qnr-B4 (1)	
	Qnr-S1 (5)	
	Qnr-S2 (2)	
	2 *gyrA* mutations + 1 *parC* mutation (109)	High-level resistance (271)
	2 *gyrA* mutations + 1 *parC* mutation + AAC(6′)-Ib-*cr* (21)	
	2 *gyrA* mutations + 1 *parC* mutation + AAC(6′)-Ib-*cr* + 1 *marR* mutation (1)	
	2 *gyrA* mutations + 1 *parC* mutation + OqxAB (2)	
	2 *gyrA* mutations + 1 *parC* mutation + OqxAB + 2 *marR* mutations (1)	
	2 *gyrA* mutations + 1 *parC* mutation + Qep-A2 (1)	
	2 *gyrA* mutations + 1 *parC* mutation + Qep-A4 (1)	
	2 *gyrA* mutations + 1 *parC* mutation + Qnr-B4 (2)	
	2 *gyrA* mutations + 1 *parC* mutation + Qnr-S1 (4)	
	2 *gyrA* mutations + 1 *parC* mutation + Qnr-S1 + 2 *marR* mutations (1)	
	2 *gyrA* mutations + 1 *parC* mutation + Qnr-S2 (1)	
	2 *gyrA* mutations + 1 *parC* mutation + 1 *marR* mutations (2)	
	2 *gyrA* mutations + 1 *parC* mutation + 2 *marR* mutations (10)	
	2 *gyrA* mutations + 1 *parC* mutation + 1 *soxR* mutation (1)	
	2 *gyrA* mutations + 2 *parC* mutations (69)	
	2 *gyrA* mutations + 2 *parC* mutations + 1 *marR* mutation (2)	
	2 *gyrA* mutations + 2 *parC* mutations + 2 *marR* mutations (2)	

		2 *gyrA* mutations + 2 *parC* mutations + 1 *acrR* mutation (3)
		2 *gyrA* mutations + 2 *parC* mutations + AAC(6′)-Ib (1)
		2 *gyrA* mutations + 2 *parC* mutations + AAC(6′)-Ib-*cr* (34)
		2 *gyrA* mutations + 2 *parC* mutations + AAC(6′)-Ib + OqxAB (1)
		2 *gyrA* mutations + 2 *parC* mutations + Qnr-D2 + 2 *marR* mutations (1)
		2 *gyrA* mutations + 1 *parC* mutation + 1 *gyrB* mutation + 2 *marR* mutations (1)

N, number; WGS, whole-genome sequencing; CIP, ciprofloxacin

Table S5. Genotypes vs. QUIRMIA-based resistance phenotypes

WGS-based resistotype	Resistance mechanism(s) (N. of isolates)	Predicted phenotype	N. of isol.	Genotype/ phenotype concord-ance (n., %)	Genotype/ phenotype discord-ance(n., %)	Discrepant phenotypes	Discre-pant number	Clinical impact
**Wild-type (WT)**	No resistance mechanism (140)	NAL ≥ 19 mm	140	138 (98.6%)	2 (1.4%)	**NAL < 19 mm**		
		NOR ≥ 18 mm				NOR ≥ 18 mm	**1,2**	**mE**
**Low-Level Resistance (R1)**	1 *gyrA* mutation (101)	NAL < 19 mm	142	135 (95.1%)	7 (4.9%)	**NAL ≥ 19 mm**		
	1 *gyrA* mutation + 1 *parC* mutation (9)	NOR ≥ 18 mm				NOR ≥ 18 mm	3,4,5,6	-
	1 *gyrA* mutation + Qnr-A1 (1)					NAL < 19 mm		
	1 *gyrA* mutation + Qnr-S1 (5)					**NOR < 18 mm**	**7,8,9**	**mE**
	1 *gyrA* mutation + Qnr-B4 (3)							
	1 *gyrA* mutation + AAC(6')-Ib-*cr* (1)							
	1 *gyrA* mutation + 2 *marR* mutations (5)							
	2 *gyrA* mutations (4)							
	AAC(6')-Ib-cr (2)							
	Qnr-S1 (6)							
	Qnr-S1 + 2 *marR* mutations (1)							
	Qnr-S2 (2)							
	Qnr-B19 (1)							
	OqxB + 2 *marR* mutations (1)							
**High-level resistance (R2)**	2 *gyrA* mutations + 1 *parC* mutation + 1 *gyrB* mutation + 2 *marR* mutations (1)	NAL < 19 mm	271	269 (99.3%)	2 (0.7%)	NAL < 19 mm	10,11	-
	2 *gyrA* mutations + 1 *parC* mutation (109)	NOR < 18 mm				**NOR ≥ 18 mm**		

2 *gyrA* mutations + 1 *parC* mutation + AAC(6')-Ib-*cr* (21)				
2 *gyrA* mutations + 1 *parC* mutation + AAC(6')-Ib-*cr* + 1 *marR* mutation (1)				
2 *gyrA* mutations + 1 *parC* mutation + OqxAB (2)				
2 *gyrA* mutations + 1 *parC* mutation + OqxAB + 2 *marR* mutations (1)				
2 *gyrA* mutations + 1 *parC* mutation + Qep-A2 (1)				
2 *gyrA* mutations + 1 *parC* mutation + Qep-A4 (1)				
2 *gyrA* mutations + 1 *parC* mutation + Qnr-B4 (2)				
2 *gyrA* mutations + 1 *parC* mutation + Qnr-S1 (4)				
2 *gyrA* mutations + 1 *parC* mutation + Qnr-S1 + 2 *marR* mutations (1)				
2 *gyrA* mutations + 1 *parC* mutation + Qnr-S2 (1)				
2 *gyrA* mutations + 1 *parC* mutation + 1 *marR* mutations (2)				
2 *gyrA* mutations + 1 *parC* mutation + 2 *marR* mutations (10)				
2 *gyrA* mutations + 1 *parC* mutation + 1 *soxR* mutation (1)				
2 *gyrA* mutations + 2 *parC* mutations (69)				
2 *gyrA* mutations + 2 *parC* mutations + 1 *marR* mutation (2)				
2 *gyrA* mutations + 2 *parC* mutations + 2 *marR* mutations (2)				
2 *gyrA* mutations + 2 *parC* mutations + 1 *acrR* mutation (3)				
2 *gyrA* mutations + 2 *parC* mutations + AAC(6')-Ib (1)				
2 *gyrA* mutations + 2 *parC* mutations + AAC(6')-Ib-*cr* (34)				
2 *gyrA* mutations + 2 *parC* mutations + AAC(6')-Ib + OqxAB (1)				
2 *gyrA* mutations + 2 *parC* mutations + Qnr-D2 + 2 *marR* mutations (1)				
**Total**	**553**	**542 (98%)**	**11 (2 %)**	**5 mE**

N., number; NAL, nalidixic acid; NOR, norfloxacin; mE, minor error.

Table S6. Discrepancy table: WGS-based classification

Discrepant number	ST	NAL (mm)	NOR (mm)	CIP (mm)	LEV (mm)	Resistance mechanism(s)	WGS-based resistotype	Observed resistotype	Clinical impact
1	Not found	13	27	**23**	**22**	-	Wild-type	Low-level resistance	mE
2	336	15	23	**22**	**25**	-	Wild-type	Low-level resistance	mE
3	141	27	31	**33**	**34**	*aac*(6')-Ib-cr	Low-level resistance	Wild-type	-
4	Not found	22	35	**38**	**36**	*aac*(6')-Ib-cr	Low-level resistance	Wild-type	-
5	1421	21	24	**24**	**25**	*qnr*S1	Low-level resistance	Wild-type	-
6	Not found	21	33	**36**	**35**	*oqx*B, *marR* Y137H/G103S	Low-level resistance	Wild-type	-
7	131	6	17	**20**	**21**	*gyrA* S83L	Low-level resistance	High-level resistance	mE
8	Not found	6	16	**18**	**16**	*gyrA* S83L, *qnr*A1	Low-level resistance	High-level resistance	mE
9	73	6	14	**18**	**20**	*gyrA* S83L, *parC* S80I	Low-level resistance	High-level resistance	mE
10	Not found	10	21	**19**	**17**	*gyrA* S83L/D87Y, *parC* S80R, *oqx*AB	High-level resistance	Low-level resistance	-
11	162	6	19	**18**	**19**	*gyrA* S83L/D87N, *parC* S80I	High-level resistance	Low-level resistance	-

Phenotypes responsible for the discrepancy are highlighted in red. Inhibition zone diameters highlighted in black bold were included for completion but were not used for the phenotype-based classification.

ST, Sequence type; NAL, Nalidixic acid (30 µg); NOR, Norfloxacin (10 µg); CIP, Ciprofloxacin (5 µg); LEV, Levofloxacin (5 µg); mE, minor Error; vME, very major error.

Table S7. Genotypes vs. EUCAST-based categorization

WGS-based resistotype	Resistance mechanism(s) (N. of isolates)	Observed phenotype	EUCAST-based categorization	N. of isolates
**Wild-type (WT)**	No resistance mechanism (140)	CIP ≥ 25 mm	Susceptible	138 (98.6%)
		CIP < 25 mm	Resistant	2 (1.4%)
**Low-Level Resistance (R1)**	1 *gyrA* mutation (52)	CIP ≥ 25 mm	Susceptible	69 (48.6%)
	2 *gyrA* mutations (1)			
	1 *gyrA* mutation + 1 *parC* mutation (2)			
	1 *gyrA* mutation + Qnr-S1 (1)			
	1 *gyrA* mutation + Qnr-B4 (2)			
	1 *gyrA* mutation + 2 *marR* mutations (5)			
	AAC(6')-Ib-*cr* (2)			
	Qnr-B19 (1)			
	Qnr-S1 (1)			
	Qnr-S1+ 2 *marR* mutations (1)			
	OqxB + 2 *marR* mutations (1)			
	1 *gyrA* mutation (49)	CIP < 25 mm	Resistant	73 (51.4%)
	2 *gyrA* mutations (3)			
	1 *gyrA* mutation + 1 *parC* mutation (7)			
	1 *gyrA* mutation + Qnr-S1 (4)			
	AAC(6')-Ib-*cr* (1)			
	Qnr-A1 (1)			
	Qnr-B4 (1)			

	Qnr-S1 (5)			
	Qnr-S2 (2)			
**High-level resistance (R2)**	2 *gyrA* mutations + 1 *parC* mutation + 1 *gyrB* mutation + 2 *marR* mutations (1)	CIP < 25 mm	Resistant	271 (100%)
	2 *gyrA* mutations + 1 *parC* mutation (109)			
	2 *gyrA* mutations + 1 *parC* mutation + AAC(6')-Ib-*cr* (21)			
	2 *gyrA* mutations + 1 *parC* mutation + AAC(6')-Ib-*cr* + 1 *marR* mutation (1)			
	2 *gyrA* mutations + 1 *parC* mutation + OqxAB (2)			
	2 *gyrA* mutations + 1 *parC* mutation + OqxAB + 2 *marR* mutations (1)			
	2 *gyrA* mutations + 1 *parC* mutation + Qep-A2 (1)			
	2 *gyrA* mutations + 1 *parC* mutation + Qep-A4 (1)			
	2 *gyrA* mutations + 1 *parC* mutation + Qnr-B4 (2)			
	2 *gyrA* mutations + 1 *parC* mutation + Qnr-S1 (4)			
	2 *gyrA* mutations + 1 *parC* mutation + Qnr-S1 + 2 *marR* mutations (1)			
	2 *gyrA* mutations + 1 *parC* mutation + Qnr-S2 (1)			
	2 *gyrA* mutations + 1 *parC* mutation + 1 *marR* mutations (2)			
	2 *gyrA* mutations + 1 *parC* mutation + 2 *marR* mutations (10)			
	2 *gyrA* mutations + 1 *parC* mutation + 1 *soxR* mutation (1)			
	2 *gyrA* mutations + 2 *parC* mutations (69)			
	2 *gyrA* mutations + 2 *parC* mutations + 1 *marR* mutation (2)			
	2 *gyrA* mutations + 2 *parC* mutations + 2 *marR* mutations (2)			
	2 *gyrA* mutations + 2 *parC* mutations + 1 *acrR* mutation (3)			
	2 *gyrA* mutations + 2 *parC* mutations + AAC(6')-Ib (1)			
	2 *gyrA* mutations + 2 *parC* mutations + AAC(6')-Ib-*cr* (34)			

	2 *gyrA* mutations + 2 *parC* mutations + AAC(6')-Ib + OqxAB (1)	
	2 *gyrA* mutations + 2 *parC* mutations + Qnr-D2 + 2 *marR* mutations (1)	
**Total**		**553**

N., number; CIP, ciprofloxacin.

Table S8. Clinical isolates used in this study

N.	NAL (mm)	CIP (mm)	NOR (mm)	LEV (mm)	Mutation							Resistotype based on CIP pheno-type	Resistotype based on NOR phe-notype	Resistotype based on LEV pheno-type	Resistotype based on genotype	Beta-lactamase production
					GyrA	ParC	Others	MarR	AcrR	SoxR	RpoB					
1	7	23	22	30	S83L		Aac(6')-Ib-cr					R1	R1	R1	R1	ESBL
2	6	6	6	10	S83L/D87N	S80I		G103S, Y137H				R2	R2	R2	R2	Carba
3	7	22	24	22	S83L							R1	R1	R1	R1	ESBL
4	6	24	29	24	S83L							R1	R1	R1	R1	WT
5	7	25	26	22	S83L							R1	R1	R1	R1	WT
6	7	22	26	21	S83L							R1	R1	R1	R1	ESBL
7	6	6	6	10	S83L/D87N	S80I/E84V						R2	R2	R2	R2	ESBL
8	26	36	35	35								WT	WT	WT	WT	WT
9	6	25	23	23	D87G							R1	R1	R1	R1	WT
10	6	13	11	11	S83L/D87N	S80I						R2	R2	R2	R2	ESBL
11	6	6	6	10	S83L/D87N	S80I/E84V						R2	R2	R2	R2	WT
12	6	6	6	6	S83L/D87N	S80I/E84V						R2	R2	R2	R2	ESBL
13	6	6	6	6	S83L/D87N	S80I/E84V			E67trunc			R2	R2	R2	R2	WT
14	6	6	6	6	S83L/D87Y	S80I/E84V			H115Y			R2	R2	R2	R2	WT
15	6	6	6	13	S83L/D87N	S80I						R2	R2	R2	R2	WT
16	6	9	6	11	S83L/D87N	S80I						R2	R2	R2	R2	WT
17	6	6	6	6	S83L/D87N	S80I						R2	R2	R2	R2	ESBL
18	6	6	6	6	S83L/D87N	S80I						R2	R2	R2	R2	WT
19	26	30	28	28								WT	WT	WT	WT	WT
20	6	24	22	23	S83L							R1	R1	R1	R1	WT
21	6	6	6	6	S83L/D87N	S80I						R2	R2	R2	R2	WT
22	6	6	6	6	S83L/D87N	S80I	Qep-A2					R2	R2	R2	R2	ESBL
23	27	28	26	29								WT	WT	WT	WT	WT
24	6	15	12	17	S83L/D87N	S80I						R2	R2	R2	R2	WT
25	27	34	31	36								WT	WT	WT	WT	WT
26	7	19	28	19	S83L							R1	R1	R1	R1	WT
27	6	6	6	9	S83L/D87N	S80I						R2	R2	R2	R2	WT
28	6	6	6	10	S83L/D87N	S80I/E84V						R2	R2	R2	R2	WT
29	6	6	6	10	S83L/D87N	S80I/E84V						R2	R2	R2	R2	WT

30	24	30	23	25								WT	WT	WT	WT	WT
31	6	6	6	9	S83L/D87N	S80I/E84V						R2	R2	R2	R2	WT
32	21	33	30	30								WT	WT	WT	WT	WT
33	24	31	29	29								WT	WT	WT	WT	ESBL
34	6	6	6	6	S83L/D87N	S80I						R2	R2	R2	R2	ESBL
35	6	6	6	9	S83L/D87N	S80I/E84V			K55trunc			R2	R2	R2	R2	ESBL
36	6	26	25	23	S83L							R1	R1	R1	R1	WT
37	28	37	32	34								WT	WT	WT	WT	WT
38	6	6	6	8	S83L/D87N	S80I						R2	R2	R2	R2	WT
39	6	6	6	6	S83L/D87N	S80I/E84V						R2	R2	R2	R2	WT
40	6	6	6	10	S83L/D87N	S80I						R2	R2	R2	R2	WT
41	25	37	37	35								WT	WT	WT	WT	WT
42	29	37	36	36								WT	WT	WT	WT	WT
43	9	23	21	24	S83L	S80I						R1	R1	R1	R1	WT
44	6	6	6	11	S83L/D87N	S80I						R2	R2	R2	R2	WT
45	6	6	6	11	S83L/D87N	S80I						R2	R2	R2	R2	WT
46	26	37	35	37								WT	WT	WT	WT	WT
47	6	6	6	11	S83L/D87N	S80I/E84V						R2	R2	R2	R2	ESBL
48	6	6	6	6	S83L/D87N	S80I						R2	R2	R2	R2	WT
49	6	6	6	6	S83L/D87N	S80I/E84G						R2	R2	R2	R2	WT
50	6	6	6	6	S83L/D87N	S80I						R2	R2	R2	R2	WT
51	6	6	6	6	S83L/D87N	S80I/E84G						R2	R2	R2	R2	WT
52	26	35	33	35								WT	WT	WT	WT	WT
53	6	6	6	8	S83L/D87N	S80I/E84V						R2	R2	R2	R2	ESBL
54	6	6	6	6	S83L/D87N	S80I/E84V						R2	R2	R2	R2	ESBL
55	6	6	6	6	S83L/D87N	S80I						R2	R2	R2	R2	ESBL
56	24	36	34	34								WT	WT	WT	WT	WT
57	6	18	19	19	S83L/D87N	S80I						R1	R1	R1	R2	WT
58	6	6	6	10	S83L/D87N	S80I						R2	R2	R2	R2	WT
59	6	6	6	11	S83L/D87N	S80I/E84V						R2	R2	R2	R2	ESBL
60	6	28	24	25	S83L/D87G							R1	R1	R1	R1	WT
61	6	6	6	8	S83L/D87N	S80I/E84V						R2	R2	R2	R2	ESBL
62	30	37	37	37								WT	WT	WT	WT	WT

63	26	31	31	30								WT	WT	WT	WT	WT
64	15	28	29	26			Qnr-S1					R1	R1	R1	R1	WT
65	21	31	29	29								WT	WT	WT	WT	WT
66	6	12	12	14	S83L/D87N	S80I						R2	R2	R2	R2	WT
67	6	6	6	9	S83L/D87N	S80I						R2	R2	R2	R2	WT
68	6	6	6	6	S83L/D87N	E84K						R2	R2	R2	R2	WT
69	6	27	24	27	S83L							R1	R1	R1	R1	ESBL
70	6	6	6	6	S83L/D87N	S80I						R2	R2	R2	R2	WT
71	6	23	23	21	S83L/D87G							R1	R1	R1	R1	WT
72	26	34	36	34								WT	WT	WT	WT	WT
73	6	6	6	12	S83L/D87N	S80I						R2	R2	R2	R2	WT
74	6	15	13	15	S83L/D87N	S80I						R2	R2	R2	R2	WT
75	6	11	6	15	S83L/D87N	S80I						R2	R2	R2	R2	WT
76	6	19	20	19	S83L		Qnr-S1					R1	R1	R1	R1	WT
77	6	6	6	10	S83L/D87N	S80I						R2	R2	R2	R2	ESBL
78	27	38	35	37								WT	WT	WT	WT	ESBL
79	6	6	6	12	S83L/D87N	S80I/E84V						R2	R2	R2	R2	ESBL
80	6	6	6	12	S83L/D87N	S80I						R2	R2	R2	R2	WT
81	6	6	6	9	S83L/D87N	S80I/E84V						R2	R2	R2	R2	ESBL
82	6	13	10	14	S83L/D87N	S80I						R2	R2	R2	R2	ESBL
83	6	14	10	14	S83L/D87H	S80R						R2	R2	R2	R2	WT
84	23	30	31	31								WT	WT	WT	WT	WT
85	6	6	6	8	S83L/D87N	S80I/E84G						R2	R2	R2	R2	WT
86	6	6	6	13	S83L/D87N	S80I/E84V	AAC(6')-Ib-cr					R2	R2	R2	R2	WT
87	6	6	6	9	S83L/D87N	S80I	AAC(6')-Ib-cr					R2	R2	R2	R2	WT
88	6	6	6	9	S83L/D87N	S80I	AAC(6')-Ib-cr					R2	R2	R2	R2	WT
89	6	8	6	10	S83L/D87N	S80I/E84V						R2	R2	R2	R2	WT
90	6	6	6	6	S83L/D87N	S80I/E84V	AAC(6')-Ib-cr					R2	R2	R2	R2	WT
91	25	35	36	33								WT	WT	WT	WT	WT
92	6	26	26	27	S83L							R1	R1	R1	R1	WT
93	6	6	6	12	S83L/D87N	S80I/E84V	AAC(6')-Ib-cr					R2	R2	R2	R2	ESBL
94	6	26	27	25	S83L		Qnr-B4					R1	R1	R1	R1	ESBL
95	6	26	27	25	S83L		Qnr-B4					R1	R1	R1	R1	ESBL

96	30	33	36	34								WT	WT	WT	WT	WT
97	6	6	6	6	S83L/D87N	S80I/E84V	AAC(6')-Ib-cr					R2	R2	R2	R2	WT
98	6	6	6	10	S83L/D87N	S80I/E84V	AAC(6')-Ib-cr					R2	R2	R2	R2	WT
99	6	11	12	14	S83L/D87N	S80I						R2	R2	R2	R2	ESBL
100	6	9	10	9	S83L/D87N	S80I	Qnr-S1					R2	R2	R2	R2	ESBL
101	22	32	29	32								WT	WT	WT	WT	ESBL
102	22	32	29	32								WT	WT	WT	WT	ESBL
103	6	6	6	6	S83L/D87N	S80I						R2	R2	R2	R2	WT
104	6	12	10	16	S83L/D87N	S80I						R2	R2	R2	R2	WT
105	6	24	19	23	S83L							R1	R1	R1	R1	ESBL
106	6	24	20	23	S83L							R1	R1	R1	R1	ESBL
107	6	13	6	15	S83L/D87N	S80I						R2	R2	R2	R2	ESBL
108	6	11	10	11	S83L/D87N	S80I						R2	R2	R2	R2	WT
109	6	16	17	21	S83L/D87N	S80I						R2	R2	R1	R2	WT
110	6	26	27	26	S83L							R1	R1	R1	R1	WT
111	6	6	6	6	S83L/D87N	S80I	Qnr-B4					R2	R2	R2	R2	ESBL
112	6	6	6	6	S83L/D87N	S80I	AAC(6')-Ib-cr					R2	R2	R2	R2	WT
113	6	23	22	23	S83L							R1	R1	R1	R1	WT
114	6	35	12	34	S83L/D87N	S80I						R2	R2	R2	R2	WT
115	6	21	19	24	S83L							R1	R1	R1	R1	WT
116	28	33	31	33								WT	WT	WT	WT	WT
117	22	30	29	28								WT	WT	WT	WT	WT
118	27	33	31	34			AAC(6')-Ib-cr					WT	WT	WT	R1	WT
119	6	7	6	16	S83L/D87N	S80I				R20L		R2	R2	R2	R2	ESBL
120	6	12	12	14	S83L/D87N	S80I						R2	R2	R2	R2	WT
121	6	6	6	6	S83L/D87N	S80I/E84K						R2	R2	R2	R2	WT
122	6	6	6	9	S83L/D87N	S80I/E84V	AAC(6')-Ib-cr					R2	R2	R2	R2	ESBL
123	6	6	6	11	S83L/D87N	S80I/E84V	AAC(6')-Ib-cr					R2	R2	R2	R2	WT
124	13	26	29	30	D87Y							R1	R1	R1	R1	WT
125	6	29	28	29	S83L							R1	R1	R1	R1	WT
126	6	6	6	12	S83L/D87N	S80I/E84V	AAC(6')-Ib-cr					R2	R2	R2	R2	WT
127	6	8	6	13	S83L/D87N	S80I						R2	R2	R2	R2	WT
128	26	36	32	33								WT	WT	WT	WT	WT

129	24	35	34	30								WT	WT	WT	WT	WT
130	24	40	36	34								WT	WT	WT	WT	WT
131	6	28	27	27	S83L							R1	R1	R1	R1	WT
132	6	6	6	6	S83L/D87N	S80I/E84V						R2	R2	R2	R2	WT
133	6	6	6	10	S83L/D87N	S80I/E84V						R2	R2	R2	R2	ESBL
134	26	34	35	36								WT	WT	WT	WT	WT
135	6	12	10	15	S83L/D87N	S80I						R2	R2	R2	R2	WT
136	6	9	6	11	S83L/D87N	S80I						R2	R2	R2	R2	WT
137	6	9	6	9	S83L/D87N	S80I						R2	R2	R2	R2	WT
138	6	25	23	26	S83L	S80I						R1	R1	R1	R1	WT
139	6	28	28	28	S83L							R1	R1	R1	R1	WT
140	6	6	6	6	S83L/D87N	S80I/E84K						R2	R2	R2	R2	WT
141	6	6	6	6	S83L/D87N	S80I/E84K						R2	R2	R2	R2	WT
142	6	6	6	7	S83L/D87N	S80I/E84V						R2	R2	R2	R2	WT
143	6	6	6	8	S83L/D87N	S80I/E84V	AAC(6')-Ib-cr					R2	R2	R2	R2	WT
144	6	6	6	9	S83L/D87N	S80I/E84V	AAC(6')-Ib-cr					R2	R2	R2	R2	ESBL
145	6	14	6	19	S83L/D87N	S80I						R2	R2	R1	R2	WT
146	6	30	28	28	S83L							R1	R1	R1	R1	WT
147	6	8	6	11	S83L/D87N	S80I						R2	R2	R2	R2	WT
148	29	36	32	35								WT	WT	WT	WT	WT
149	6	6	6	10	S83L/D87N	**S80I**						R2	R2	R2	R2	WT
150	6	22	22	22	S83L							R1	R1	R1	R1	WT
151	6	9	6	16	S83L/D87N	**S80I**						R2	R2	R2	R2	WT
152	27	33	31	32								WT	WT	WT	WT	WT
153	6	24	26	25	S83L							R1	R1	R1	R1	ESBL
154	6	9	6	13	S83L/D87N	S80I						R2	R2	R2	R2	ESBL
155	23	33	31	31								WT	WT	WT	WT	WT
156	30	40	36	37								WT	WT	WT	WT	WT
157	6	27	29	28	S83L							R1	R1	R1	R1	WT
158	6	6	6	9	S83L/D87N	S80I	AAC(6')-Ib-cr					R2	R2	R2	R2	ESBL
159	6	6	6	16	S83L/D87N	**S80I**						R2	R2	R2	R2	WT
160	26	31	28	29								WT	WT	WT	WT	WT
161	6	24	23	23	S83L							R1	R1	R1	R1	WT

162	6	30	28	28	S83L							R1	R1	R1	R1	WT
163	6	6	6	7	S83L/D87N	S80I/E84V						R2	R2	R2	R2	ESBL
164	6	6	6	6	S83L/D87N	S80I/E84G	AAC(6')-Ib					R2	R2	R2	R2	WT
165	6	6	6	9	S83L/D87N	S80I						R2	R2	R2	R2	WT
166	6	6	6	7	S83L/D87N	S80I						R2	R2	R2	R2	WT
167	24	31	29	32								WT	WT	WT	WT	WT
168	26	35	34	35								WT	WT	WT	WT	WT
169	6	6	6	10	S83L/D87N	S80I/E84V	AAC(6')-Ib-cr					R2	R2	R2	R2	WT
170	26	31	33	30								WT	WT	WT	WT	WT
171	6	30	27	26	S83L							R1	R1	R1	R1	ESBL
172	23	34	34	31								WT	WT	WT	WT	WT
173	6	6	6	6	S83L/D87N	S80I/E84V	AAC(6')-Ib-cr					R2	R2	R2	R2	ESBL
174	25	32	29	30								WT	WT	WT	WT	WT
175	26	34	30	33								WT	WT	WT	WT	WT
176	30	35	32	34								WT	WT	WT	WT	WT
177	24	37	36	36								WT	WT	WT	WT	WT
178	6	17	15	19	S83L/D87N	S80I						R2	R2	R1	R2	WT
179	23	32	31	31								WT	WT	WT	WT	WT
180	25	37	32	34								WT	WT	WT	WT	WT
181	6	6	6	6	S83L/D87N	S83L/D87N		V84G				R2	R2	R2	R2	WT
182	29	35	33	35								WT	WT	WT	WT	WT
183	28	34	32	34								WT	WT	WT	WT	WT
184	14	30	29	27			Qnr-B19					R1	R1	R1	R1	ESBL
185	6	6	6	12	S83L/D87N	S80I/E84V	AAC(6')-Ib-cr					R2	R2	R2	R2	WT
186	6	27	26	27	S83L							R1	R1	R1	R1	WT
187	6	6	6	10	S83L/D87N	S80I/E84V						R2	R2	R2	R2	WT
188	6	6	6	8	S83L/D87Y	S80I/E84G						R2	R2	R2	R2	ESBL
189	6	14	12	16	S83L/D87N	S80I						R2	R2	R2	R2	WT
190	6	6	6	6	S83L/D87N	S80I/E84G	AAC(6')-Ib/ OqxAB					R2	R2	R2	R2	ESBL
191	6	15	10	15	S83L/D87Y	S80I						R2	R2	R2	R2	WT
192	6	31	28	29	S83L							R1	R1	R1	R1	WT
193	6	17	13	17	S83L/D87N	S80I						R2	R2	R2	R2	WT
194	6	6	6	6	S83L/D87N	S80I/E84V	AAC(6')-Ib-cr					R2	R2	R2	R2	WT

195	24	30	26	28								WT	WT	WT	WT	WT
196	26	36	34	34								WT	WT	WT	WT	WT
197	26	38	36	36								WT	WT	WT	WT	ESBL
198	6	6	6	9	S83L/D87N	S80I						R2	R2	R2	R2	WT
199	25	33	33	33								WT	WT	WT	WT	WT
200	27	35	34	34								WT	WT	WT	WT	WT
201	6	6	6	6	S83L/D87Y	S80I/E84G						R2	R2	R2	R2	ESBL
202	26	34	33	32								WT	WT	WT	WT	WT
203	6	6	6	9	S83L/D87N	S80I						R2	R2	R2	R2	WT
204	6	6	6	6	S83L/D87Y	S80I/E84G						R2	R2	R2	R2	WT
205	6	6	6	8	S83L/D87N	S80I	AAC(6')-Ib-cr					R2	R2	R2	R2	WT
206	6	6	7	7	S83L/D87N	S80I	AAC(6')-Ib-cr					R2	R2	R2	R2	WT
207	6	6	6	10	S83L/D87N	S80I/E84V						R2	R2	R2	R2	ESBL
208	6	6	6	9	S83L/D87Y	S80I/E84V	AAC(6')-Ib-cr					R2	R2	R2	R2	WT
209	6	6	6	8	S83L/D87Y	S80I/E84G						R2	R2	R2	R2	WT
210	22	37	34	33								WT	WT	WT	WT	WT
211	6	6	6	8	S83L/D87N	S80I/E84V	AAC(6')-Ib-cr					R2	R2	R2	R2	ESBL
212	6	6	6	12	S83L/D87N	S80I						R2	R2	R2	R2	WT
213	6	6	6	6	S83L/D87N	S80I	Qnr-B4					R2	R2	R2	R2	ESBL
214	6	22	25	22			Qnr-S1					R1	R1	R1	R1	WT
215	6	23	27	23			Qnr-S2					R1	R1	R1	R1	WT
216	6	6	6	11	S83L/D87N	S80I/E84V						R2	R2	R2	R2	ESBL
217	23	28	30	25								WT	WT	WT	WT	WT
218	6	20	24	19	S83L		Qnr-S1					R1	R1	R1	R1	ESBL
219	6	6	6	9	S83L/D87N	S80I	AAC(6')-Ib-cr					R2	R2	R2	R2	ESBL
220	28	32	29	32								WT	WT	WT	WT	WT
221	22	28	27	27								WT	WT	WT	WT	WT
222	6	6	6	9	S83L/D87N	S80I	AAC(6')-Ib-cr					R2	R2	R2	R2	WT
223	25	31	30	31								WT	WT	WT	WT	WT
224	6	6	6	6	S83L/D87N	S80I/E84V	AAC(6')-Ib-cr					R2	R2	R2	R2	ESBL
225	6	14	14	17	S83L/D87N	S80I						R2	R2	R2	R2	WT
226	26	32	28	29								WT	WT	WT	WT	WT
227	6	26	25	27	S83L							R1	R1	R1	R1	WT

228	6	6	6	6	S83L/D87N	S80I	AAC(6')-Ib-cr					R2	R2	R2	R2	ESBL
229	6	6	6	6	S83L/D87N	S80I	AAC(6')-Ib-cr					R2	R2	R2	R2	ESBL
230	6	6	6	6	S83L/D87N	S80I/E84V	AAC(6')-Ib-cr					R2	R2	R2	R2	ESBL
231	22	26	24	24								WT	WT	WT	WT	WT
232	6	6	6	6	S83L/D87N	S80I						R2	R2	R2	R2	WT
233	6	6	6	11	S83L/D87N	S80I/E84V	AAC(6')-Ib-cr					R2	R2	R2	R2	ESBL
234	7	21	22	19	D87N		Qnr-S1					R1	R1	R1	R1	WT
235	6	11	6	15	S83L/D87N	S80I						R2	R2	R2	R2	WT
236	23	30	28	30								WT	WT	WT	WT	WT
237	7	30	28	28	D87Y							R1	R1	R1	R1	WT
238	6	26	23	26	S83L							R1	R1	R1	R1	WT
239	6	6	6	10	S83L/D87N	S80I	AAC(6')-Ib-cr					R2	R2	R2	R2	WT
240	6	6	6	6	S83L/D87N	S80I						R2	R2	R2	R2	WT
241	6	6	6	10	S83L/D87N	S80I	AAC(6')-Ib-cr					R2	R2	R2	R2	WT
242	19	27	26	26								WT	WT	WT	WT	WT
243	21	24	24	25			Qnr-S1					WT	WT	WT	R1	WT
244	6	6	6	6	S83L/D87Y	S80I/E84V	AAC(6')-Ib-cr					R2	R2	R2	R2	WT
245	25	35	32	32								WT	WT	WT	WT	WT
246	6	27	26	28	S83L							R1	R1	R1	R1	WT
247	30	38	35	34								WT	WT	WT	WT	WT
248	6	6	6	11	S83L/D87Y	S80I/E84V	AAC(6')-Ib-cr					R2	R2	R2	R2	WT
249	6	12	11	8	S83L/D87N	S80I						R2	R2	R2	R2	WT
250	6	12	6	13	S83L/D87N	S80I						R2	R2	R2	R2	WT
251	21	29	27	26								WT	WT	WT	WT	WT
252	10	23	22	22			Qnr-S1					R1	R1	R1	R1	WT
253	6	6	6	11	S83L/D87N	S80I/E84V	AAC(6')-Ib-cr					R2	R2	R2	R2	ESBL
254	6	31	29	29	S83L							R1	R1	R1	R1	WT
255	6	6	6	8	S83L/D87Y	S80I/E84G						R2	R2	R2	R2	ESBL
256	6	12	13	13	S83L/D87N	S80I						R2	R2	R2	R2	WT
257	6	6	6	6	S83L/D87N	S80I						R2	R2	R2	R2	ESBL
258	21	36	34	36								WT	WT	WT	WT	WT
259	6	25	25	25	S83L							R1	R1	R1	R1	WT
260	6	24	22	23	S83L							R1	R1	R1	R1	WT

261	6	23	23	23	S83L/D87A							R1	R1	R1	R1	WT
262	24	30	30	28								WT	WT	WT	WT	WT
263	6	6	6	9	S83L/D87N	S80I/E84V						R2	R2	R2	R2	ESBL
264	27	30	29	31								WT	WT	WT	WT	WT
265	6	6	6	8	S83L/D87Y	S80I/E84G						R2	R2	R2	R2	WT
266	6	6	6	9	S83L/D87Y	S80I/E84V						R2	R2	R2	R2	WT
267	6	13	12	13	S83L/D87N	S80I						R2	R2	R2	R2	WT
268	6	6	6	6	S83L/D87N	G78D	GyrB E466D	G103S, Y137H				R2	R2	R2	R2	WT
269	27	33	32	32								WT	WT	WT	WT	WT
270	6	6	6	9	S83L/D87N	S80I/E84V	AAC(6')-Ib-cr					R2	R2	R2	R2	ESBL
271	6	6	6	9	S83L/D87Y	S80I/E84G						R2	R2	R2	R2	WT
272	6	13	11	16	S83L/D87N	S80I						R2	R2	R2	R2	WT
273	6	6	6	9	S83L/D87N	S80I	Qnr-S1					R2	R2	R2	R2	WT
274	28	37	32	33								WT	WT	WT	WT	WT
275	6	27	20	26	S83L							R1	R1	R1	R1	ESBL
276	6	27	20	26	S83L							R1	R1	R1	R1	ESBL
277	6	6	6	6	S83L/D87N	S80I/E84V						R2	R2	R2	R2	WT
278	6	6	6	11	S83L/D87N	S80I/E84V						R2	R2	R2	R2	WT
279	6	6	6	9	S83L/D87N	S80I/E84V	AAC(6')-Ib-cr					R2	R2	R2	R2	ESBL
280	6	16	15	18	S83L/D87N	S80I						R2	R2	R1	R2	WT
281	6	6	6	6	S83L/D87N	S80I/E84V	AAC(6')-Ib-cr					R2	R2	R2	R2	WT
282	23	32	29	31								WT	WT	WT	WT	WT
283	6	6	6	9	S83L/D87N	S80I						R2	R2	R2	R2	WT
284	28	40	40	40								WT	WT	WT	WT	WT
285	6	6	6	6	S83L/D87N	S80I	Qnr-S1					R2	R2	R2	R2	WT
286	6	6	6	6	S83L/D87N	S80I	AAC(6')-Ib-cr					R2	R2	R2	R2	WT
287	6	6	6	8	S83L/D87N	S80I						R2	R2	R2	R2	WT
288	9	11	9	12	S83L/D87N	E84K						R2	R2	R2	R2	WT
289	29	35	31	31								WT	WT	WT	WT	WT
290	6	30	29	27	S83L							R1	R1	R1	R1	WT
291	24	38	36	34								WT	WT	WT	WT	WT
292	6	11	10	12	S83L/D87N	S80I						R2	R2	R2	R2	WT
293	23	32	30	32								WT	WT	WT	WT	WT

294	28	34	34	31								WT	WT	WT	WT	WT
295	6	26	23	26	S83L	S80R						R1	R1	R1	R1	WT
296	6	6	6	9	S83L/D87N	S80I	AAC(6')-Ib-cr					R2	R2	R2	R2	ESBL
297	22	29	26	28								WT	WT	WT	WT	WT
298	6	6	6	9	S83L/D87N	S80I/E84V						R2	R2	R2	R2	WT
299	6	6	6	9	S83L/D87N	S80I						R2	R2	R2	R2	WT
300	23	33	29	30								WT	WT	WT	WT	WT
301	23	32	30	32								WT	WT	WT	WT	WT
302	26	34	36	36								WT	WT	WT	WT	WT
303	6	16	18	15	S83L		Qnr-S1					R2	R1	R2	R1	WT
304	6	6	6	11	S83L/D87N	S80I/E84V						R2	R2	R2	R2	ESBL
305	29	32	34	31								WT	WT	WT	WT	WT
306	6	14	11	16	S83L/D87N	S80I						R2	R2	R2	R2	WT
307	6	6	6	9	S83L/D87N	S80I/E84V						R2	R2	R2	R2	ESBL
308	6	22	22	22	S83L							R1	R1	R1	R1	WT
309	6	6	6	6	S83L/D87N	S80I/E84V						R2	R2	R2	R2	ESBL
310	6	6	6	11	S83L/D87N	S80I/E84V						R2	R2	R2	R2	ESBL
311	6	6	6	10	S83L/D87N	S80I/E84V						R2	R2	R2	R2	ESBL
312	6	6	6	9	S83L/D87N	S80I/E84G		G103S, Y137H				R2	R2	R2	R2	Carba
313	6	6	6	13	S83L/D87N	S80I/E84V	AAC(6')-Ib-cr					R2	R2	R2	R2	ESBL
314	6	6	6	6	S83L/D87N	S80I						R2	R2	R2	R2	ESBL
315	6	31	29	31	S83L							R1	R1	R1	R1	WT
316	6	6	6	14	S83L/D87N	S80I/E84V	AAC(6')-Ib-cr					R2	R2	R2	R2	ESBL
317	6	6	6	12	S83L/D87N	S80I/E84V						R2	R2	R2	R2	AmpC
318	27	39	32	35								WT	WT	WT	WT	WT
319	6	20	17	21	S83L							R1	R2	R1	R1	WT
320	6	26	24	26	S83L							R1	R1	R1	R1	ESBL
321	6	38	22	36	S83L							R1	R1	R1	R1	WT
322	6	18	20	17	S83L/D87Y							R1	R1	R2	R1	WT
323	6	18	18	17	S83L	S80I						R1	R1	R2	R1	WT
324	6	6	6	8	S83L/D87N	S80I						R2	R2	R2	R2	WT
325	29	37	34	36								WT	WT	WT	WT	ESBL
326	6	6	6	12	S83L/D87N	S80I/E84V						R2	R2	R2	R2	WT

327	6	6	6	10	S83L/D87N	S80I						R2	R2	R2	R2	WT
328	6	6	6	10	S83L/D87N	S80I/E84G						R2	R2	R2	R2	WT
329	26	31	30	29								WT	WT	WT	WT	ESBL
330	28	37	32	34								WT	WT	WT	WT	WT
331	25	28	26	27								WT	WT	WT	WT	WT
332	6	6	6	12	S83L/D87N	S80I/E84V						R2	R2	R2	R2	WT
333	26	33	29	30								WT	WT	WT	WT	WT
334	6	6	6	8	S83L/D87N	S80I/E84V	AAC(6')-Ib-cr					R2	R2	R2	R2	WT
335	6	18	14	20	S83W	S80I						R1	R2	R1	R1	WT
336	6	25	24	26	S83L							R1	R1	R1	R1	WT
337	6	6	6	12	S83L/D87N	S80I		D aa 51-63				R2	R2	R2	R2	WT
338	24	34	33	32								WT	WT	WT	WT	WT
339	24	35	33	34								WT	WT	WT	WT	WT
340	6	6	6	11	S83L/D87N	S80I	AAC(6')-Ib-cr					R2	R2	R2	R2	ESBL
341	27	31	29	30								WT	WT	WT	WT	WT
342	6	6	6	7	S83L/D87Y	S80I/E84G						R2	R2	R2	R2	WT
343	6	9	6	12	S83L/D87N	S80I						R2	R2	R2	R2	WT
344	22	38	35	36			AAC(6')-Ib-cr					WT	WT	WT	R1	WT
345	6	30	28	28	S83L							R1	R1	R1	R1	WT
346	6	23	22	23	S83L							R1	R1	R1	R1	WT
347	6	6	6	6	S83L/D87Y	S80I/E84V	AAC(6')-Ib-cr					R2	R2	R2	R2	WT
348	6	6	6	6	S83L/D87N	S80I/E84K						R2	R2	R2	R2	WT
349	6	21	20	21	S83L							R1	R1	R1	R1	WT
350	6	6	6	9	S83L/D87N	S80I/E84V						R2	R2	R2	R2	WT
351	23	27	27	25								WT	WT	WT	WT	WT
352	22	33	31	32								WT	WT	WT	WT	WT
353	6	6	6	6	S83L/D87N	S80I						R2	R2	R2	R2	ESBL
354	28	33	29	30								WT	WT	WT	WT	WT
355	26	36	35	33								WT	WT	WT	WT	WT
356	6	6	6	8	S83L/D87N	S80I/E84V						R2	R2	R2	R2	ESBL
357	6	6	6	8	S83L/D87N	S80I/E84V						R2	R2	R2	R2	WT
358	6	6	6	6	S83L/D87Y	S80I	OqxAB					R2	R2	R2	R2	ESBL
359	6	12	14	17	S83L/D87N	S80I						R2	R2	R2	R2	WT

360	10	19	21	17	S83L/D87Y	S80R	OqxAB					R1	R1	R2	R2	WT
361	6	13	12	14	S83L/D87N	S80I						R2	R2	R2	R2	WT
362	6	12	12	13	S83L/D87N	S80I						R2	R2	R2	R2	WT
363	6	6	6	11	S83L/D87N	S80I/E84V						R2	R2	R2	R2	WT
364	24	30	26	27								WT	WT	WT	WT	WT
365	6	24	22	23	S83L							R1	R1	R1	R1	WT
366	6	6	6	6	S83L/D87N	S80I						R2	R2	R2	R2	WT
367	6	6	6	6	S83L/D87N	S80I						R2	R2	R2	R2	WT
368	6	6	6	6	S83L/D87N	S80I						R2	R2	R2	R2	WT
369	28	32	32	30								WT	WT	WT	WT	WT
370	6	6	6	16	S83L/D87N	S80I/E84G						R2	R2	R2	R2	WT
371	6	6	6	10	S83L/D87N	S80I/E84V						R2	R2	R2	R2	WT
372	6	6	6	10	S83L/D87N	S80I/E84V						R2	R2	R2	R2	WT
373	6	18	16	16	S83L		Qnr-A1					R1	R2	R2	R1	ESBL
374	23	30	28	28								WT	WT	WT	WT	WT
375	6	23	19	23	S83L							R1	R1	R1	R1	WT
376	6	23	19	24	S83L							R1	R1	R1	R1	WT
377	6	23	20	22	S83L							R1	R1	R1	R1	WT
378	6	23	19	23	S83L							R1	R1	R1	R1	WT
379	6	22	19	21	S83L							R1	R1	R1	R1	WT
380	26	37	35	34								WT	WT	WT	WT	WT
381	6	6	6	6	S83L/D87N	S80I/E84G						R2	R2	R2	R2	WT
382	24	34	33	32								WT	WT	WT	WT	WT
383	26	31	35	33								WT	WT	WT	WT	WT
384	6	6	6	6	S83L/D87N	S80I						R2	R2	R2	R2	AmpC/ESBL
385	28	38	36	36								WT	WT	WT	WT	WT
386	25	29	30	29								WT	WT	WT	WT	WT
387	26	32	30	28								WT	WT	WT	WT	WT
388	6	23	6	23	S83L/D87N	S80I	AAC(6')-Ib-cr					R1	R2	R1	R2	ESBL
389	6	9	6	13	S83L/D87N	S80I						R2	R2	R2	R2	WT
390	6	24	16	24	A84P/D87G	S80I						R1	R2	R1	R2	WT
391	27	38	35	36								WT	WT	WT	WT	WT
392	6	27	29	28	D87N							R1	R1	R1	R1	WT

393	6	15	15	16	S83L/D87N	S80R						R2	R2	R2	R2	WT
394	6	6	6	11	S83L/D87N	S80I/E84V	AAC(6')-Ib-cr					R2	R2	R2	R2	WT
395	6	6	6	10	S83L/D87N	S80I						R2	R2	R2	R2	WT
396	6	11	6	11	S83L/D87N	S80I						R2	R2	R2	R2	WT
397	6	11	11	11	S83L/D87N	S80I						R2	R2	R2	R2	WT
398	6	6	6	7	S83L/D87N	S80I						R2	R2	R2	R2	AmpC/ESBL
399	6	6	6	10	S83L/D87N	S80I						R2	R2	R2	R2	WT
400	6	6	6	6	S83L/D87N	S80I/E84V						R2	R2	R2	R2	ESBL
401	10	32	31	30	D87Y							R1	R1	R1	R1	WT
402	6	24	24	24	S83L							R1	R1	R1	R1	WT
403	24	35	33	34								WT	WT	WT	WT	WT
404	21	34	31	31								WT	WT	WT	WT	WT
405	26	38	34	34								WT	WT	WT	WT	WT
406	6	6	6	12	S83L/D87N	S80I						R2	R2	R2	R2	WT
407	6	6	6	10	S83L/D87N	S80I						R2	R2	R2	R2	WT
408	6	8	6	8	S83L/D87N	S80I	Qnr-S2					R2	R2	R2	R2	WT
409	26	34	33	33								WT	WT	WT	WT	WT
410	6	6	6	10	S83L/D87N	S80I/E84V	AAC(6')-Ib-cr					R2	R2	R2	R2	ESBL
411	6	25	23	24	S83L							R1	R1	R1	R1	WT
412	6	7	6	10	S83L/D87N	S80I/E84A						R2	R2	R2	R2	WT
413	6	29	29	30	S83L							R1	R1	R1	R1	ESBL
414	6	32	27	30	S83L							R1	R1	R1	R1	WT
415	22	27	29	26								WT	WT	WT	WT	ESBL
416	6	7	6	11	S83L/D87N	S80I						R2	R2	R2	R2	WT
417	6	23	25	23	S83L	E84G						R1	R1	R1	R1	WT
418	25	38	32	37								WT	WT	WT	WT	WT
419	6	6	6	9	S83L/D87Y	S80I/E84V						R2	R2	R2	R2	WT
420	25	36	33	34								WT	WT	WT	WT	WT
421	26	37	30	33								WT	WT	WT	WT	WT
422	6	6	6	9	S83L/D87N	S80I						R2	R2	R2	R2	ESBL
423	27	33	33	33								WT	WT	WT	WT	WT
424	6	26	24	25	S83L							R1	R1	R1	R1	WT
425	6	6	6	13	S83L/D87Y	S80I/E84V						R2	R2	R2	R2	WT

426	6	6	6	9	S83L/D87N	S80I	AAC(6')-Ib-cr					R2	R2	R2	R2	ESBL
427	6	6	6	9	S83L/D87N	S80I	AAC(6')-Ib-cr					R2	R2	R2	R2	ESBL
428	26	36	33	34								WT	WT	WT	WT	WT
429	14	23	22	23			Qnr-S1					R1	R1	R1	R1	WT
430	6	31	29	32	S83L							R1	R1	R1	R1	WT
431	25	31	30	31								WT	WT	WT	WT	WT
432	6	6	6	9	S83L/D87N	S80I/E84V						R2	R2	R2	R2	WT
433	6	6	6	9	S83L/D87N	S80I/E84V						R2	R2	R2	R2	ESBL
434	6	6	6	10	S83L/D87N	S80I						R2	R2	R2	R2	WT
435	6	6	6	11	S83L/D87N	S80I/E84V						R2	R2	R2	R2	ESBL
436	6	20	23	20	S83L							R1	R1	R1	R1	AmpC
437	26	36	34	33								WT	WT	WT	WT	WT
438	27	30	30	29								WT	WT	WT	WT	AmpC
439	6	6	6	6	S83L/D87N	S80I/E84V						R2	R2	R2	R2	ESBL
440	6	6	6	6	S83L/D87N	S80I	Aac(6')-Ib-cr					R2	R2	R2	R2	WT
441	6	23	22	23	S83L							R1	R1	R1	R1	WT
442	20	28	25	26								WT	WT	WT	WT	WT
443	22	27	26	27								WT	WT	WT	WT	WT
444	6	26	22	22	S83L							R1	R1	R1	R1	WT
445	25	31	29	33								WT	WT	WT	WT	WT
446	29	33	32	33								WT	WT	WT	WT	WT
447	6	27	26	26	S83L							R1	R1	R1	R1	WT
448	6	12	14	13	S83L/D87Y	S80R						R2	R2	R2	R2	WT
449	26	36	33	32								WT	WT	WT	WT	WT
450	6	35	23	37	S83L							R1	R1	R1	R1	WT
451	6	22	26	22	S83L							R1	R1	R1	R1	WT
452	6	22	21	22	S83L							R1	R1	R1	R1	WT
453	6	22	25	21	S83L							R1	R1	R1	R1	WT
454	6	13	13	15	S83L/D87N	S80I						R2	R2	R2	R2	WT
455	6	13	10	16	S83L/D87N	S80I						R2	R2	R2	R2	ESBL
456	29	34	31	31								WT	WT	WT	WT	WT
457	6	24	22	24	S83L							R1	R1	R1	R1	WT
458	6	27	24	25	S83L							R1	R1	R1	R1	WT

459	6	6	6	9	S83L/D87N	S80I	AAC(6')-Ib-cr					R2	R2	R2	R2	WT
460	6	21	20	23	S83L	S80I						R1	R1	R1	R1	ESBL
461	6	19	20	22	S83L	S80I						R1	R1	R1	R1	ESBL
462	6	11	11	13	S83L/D87N	S80I						R2	R2	R2	R2	WT
463	6	6	6	10	S83L/D87N	S80I	AAC(6')-Ib-cr					R2	R2	R2	R2	WT
464	13	23	27	22								R1	R1	R1	WT	WT
465	6	29	27	26	S83L							R1	R1	R1	R1	WT
466	6	6	6	9	S83L/D87Y	S80I/E84G	AAC(6')-Ib-cr					R2	R2	R2	R2	ESBL
467	27	37	37	37								WT	WT	WT	WT	WT
468	6	6	6	9	S83L/D87Y	S80I/E84V						R2	R2	R2	R2	WT
469	6	6	6	10	S83L/D87N	S80I/E84V						R2	R2	R2	R2	ESBL
470	6	6	6	6	S83L/D87N	S80I						R2	R2	R2	R2	ESBL
471	6	23	23	24	S83L							R1	R1	R1	R1	WT
472	7	36	6	35	S83L/D87N	S80I						R2	R2	R2	R2	ESBL
473	7	36	6	35	S83L/D87N	S80I						R2	R2	R2	R2	ESBL
474	6	6	6	15	S83L/D87	S80R	AAC(6')-Ib-cr	E85fs				R2	R2	R2	R2	WT
475	28	34	32	32								WT	WT	WT	WT	WT
476	6	6	6	6	S83L/D87N	S80I		D92fs				R2	R2	R2	R2	WT
477	24	27	24	27								WT	WT	WT	WT	WT
478	6	29	25	28	S83L							R1	R1	R1	R1	WT
479	28	40	37	36								WT	WT	WT	WT	ESBL
480	24	29	30	31								WT	WT	WT	WT	WT
481	6	27	26	26	S83L							R1	R1	R1	R1	WT
482	28	36	35	33								WT	WT	WT	WT	WT
483	26	33	34	32								WT	WT	WT	WT	WT
484	6	6	6	9	S83L/D87N	S80I						R2	R2	R2	R2	ESBL
485	6	24	23	24	S83L							R1	R1	R1	R1	WT
486	6	6	6	12	S83L/D87N	S80I						R2	R2	R2	R2	WT
487	6	6	6	6	S83L/D87N	S80I/E84V		G69fs				R2	R2	R2	R2	WT
488	6	27	26	26	S83L							R1	R1	R1	R1	WT
489	27	33	29	32								WT	WT	WT	WT	WT
490	6	23	22	22	S83L							R1	R1	R1	R1	WT
491	25	41	37	38								WT	WT	WT	WT	WT

492	6	18	18	17	S83L							R1	R1	R2	R1	WT
493	6	21	20	18	S83L							R1	R1	R1	R1	WT
494	6	21	19	20	S83L							R1	R1	R1	R1	WT
495	6	22	19	21	S83L							R1	R1	R1	R1	WT
496	6	30	31	26	S83L							R1	R1	R1	R1	WT
497	9	30	28	30	S83L							R1	R1	R1	R1	WT
498	25	34	33	32								WT	WT	WT	WT	WT
499	26	32	31	30								WT	WT	WT	WT	ESBL
500	6	6	6	11	S83L/D87N	S80I/E84V	AAC(6')-Ib-cr					R2	R2	R2	R2	ESBL
501	24	28	26	26								WT	WT	WT	WT	WT
502	6	6	6	6	S83L/D87N	S80I						R2	R2	R2	R2	WT
503	6	6	6	6	S83L/D87N	S80I						R2	R2	R2	R2	ESBL
504	6	6	6	12	S83L/D87N	S80I/E84V	AAC(6')-Ib-cr					R2	R2	R2	R2	ESBL
505	6	22	24	22	S83L							R1	R1	R1	R1	ESBL
506	6	20	22	18	S83L		Qnr-B4					R1	R1	R1	R1	WT
507	26	29	28	30								WT	WT	WT	WT	WT
508	11	30	27	29	D87Y							R1	R1	R1	R1	WT
509	6	23	26	22	S83L							R1	R1	R1	R1	WT
510	6	19	23	19	S83L							R1	R1	R1	R1	WT
511	6	21	25	20	S83L							R1	R1	R1	R1	WT
512	6	21	24	21	S83L							R1	R1	R1	R1	WT
513	13	23	24	23			Qnr-S2					R1	R1	R1	R1	WT
514	10	23	20	23	S83L							R1	R1	R1	R1	WT
515	6	24	19	24	S83L							R1	R1	R1	R1	ESBL
516	15	22	23	25								R1	R1	R1	WT	WT
517	6	27	28	27	S83L							R1	R1	R1	R1	ESBL
518	6	24	24	26	S83L							R1	R1	R1	R1	AmpC
519	6	6	6	6	S83L/D87N	S80I		Y137H, G103S				R2	R2	R2	R2	Carba
520	7	32	28	30	S83L			G103S, Y137H				R1	R1	R1	R1	Carba
521	26	33	32	31				Y137H, S3N, G103S				WT	WT	WT	WT	Carba
522	20	29	27	27				Y137H, G103S				WT	WT	WT	WT	Carba
523	6	23	22	24	S83L							R1	R1	R1	R1	WT
524	6	6	6	6	S83L/D87N	S80I	OqxB	Y137H, G103S				R2	R2	R2	R2	Carba

525	6	6	6	6	S83L/D87N	S80I		G103S, Y137H				R2	R2	R2	R2	Carba
526	14	21	22	21			Qnr-S1					R1	R1	R1	R1	ESBL
527	6	26	27	23	S83L							R1	R1	R1	R1	ESBL
528	15	22	23	22	S83L							R1	R1	R1	R1	WT
529	6	22	25	22	S83L							R1	R1	R1	R1	ESBL
530	10	25	23	26	S83L		Qnr-S1					R1	R1	R1	R1	ESBL
531	6	6	6	7	S83L/D87N	S80I	QepA4					R2	R2	R2	R2	Carba
532	6	27	20	27	S83L							R1	R1	R1	R1	WT
533	6	6	6	8	S83L/D87N	S80I		G103S, Y137H				R2	R2	R2	R2	Carba
534	6	27	27	26	S83L							R1	R1	R1	R1	ESBL
535	14	26	23	25			QnrS1	S3N, Y137H				R1	R1	R1	R1	Carba
536	21	36	33	35			OqxB	Y137H, G103S				WT	WT	WT	R1	Carba
537	6	6	6	12	S83L/D87N	S80I/E84G		G103S, Y137H				R2	R2	R2	R2	Carba
538	6	6	6	6	S83L/D87N	S80I		G103S, Y137H				R2	R2	R2	R2	Carba
539	6	25	24	24	S83L			Y137H, G103S				R1	R1	R1	R1	Carba
540	6	22	23	22	S83L							R1	R1	R1	R1	WT
541	6	21	22	22	S83L	E84G						R1	R1	R1	R1	ESBL
542	6	27	23	29	S83L			G103S, Y137H				R1	R1	R1	R1	Carba
543	6	6	6	6	S83L/D87N	S80I		Y137H, G103S				R2	R2	R2	R2	Carba
544	6	6	6	10	S83L/D87N	S80I		G103S, Y137H				R2	R2	R2	R2	Carba
545	6	6	6	11	S83L/D87N	S80I	Qnr-S1					R2	R2	R2	R2	Carba
546	6	6	6	11	S83L/D87N	S80I	Qnr-S1	Y137H, G103S				R2	R2	R2	R2	Carba
547	6	28	26	25	S83L			Y137H, G103S				R1	R1	R1	R1	Carba
548	6	6	6	8	S83L/D87N	S80I/E84G	QnrD2	G103S, Y137H				R2	R2	R2	R2	Carba
549	6	11	9	13	S83L/D87N	S80I		Y137H, G103S				R2	R2	R2	R2	Carba
550	6	27	28	26	S83L			Y137H, G103S				R1	R1	R1	R1	Carba
551	26	32	30	35				Y137H, G103S				WT	WT	WT	WT	Carba
552	6	6	6	11	S83L/D87N	S80I		Y137H, G103S				R2	R2	R2	R2	Carba
553	6	6	6	13	S83L/D87N	S80I		Y137H, G103S				R2	R2	R2	R2	Carba

ST, Sequence type; NAL, Nalidixic acid (30 µg); NOR, Norfloxacin (10 µg); CIP, Ciprofloxacin (5 µg); LEV, Levofloxacin (5 µg); R1, low-level-resistance; R2, high-level-resistance; WT, no ESBL/AmpC/carbapenemase producer.
